# Robust multi-input multi-output adaptive fuzzy terminal sliding mode control of deep brain stimulation in Parkinson’s disease: a simulation study

**DOI:** 10.1038/s41598-021-00365-9

**Published:** 2021-10-27

**Authors:** Ehsan Rouhani, Yaser Fathi

**Affiliations:** 1 Department of Electrical and Computer Engineering, Isfahan University of Technology, 84156-83111 Isfahan, Iran; 2 Department of Biomedical Engineering, School of Electrical Engineering, Iran University of Science and Technology, Tehran, Iran

**Keywords:** Biomedical engineering, Computer modelling, Control theory, Differential equations, Nonlinear dynamics, Robustness, Dynamical systems

## Abstract

Deep brain stimulation (DBS) has become an effective therapeutic solution for Parkinson’s disease (PD). Adaptive closed-loop DBS can be used to minimize stimulation-induced side effects by automatically determining the stimulation parameters based on the PD dynamics. In this paper, by modeling the interaction between the neurons in populations of the thalamic, the network-level modulation of thalamic is represented in a standard canonical form as a multi-input multi-output (MIMO) nonlinear first-order system with uncertainty and external disturbances. A class of fast and robust MIMO adaptive fuzzy terminal sliding mode control (AFTSMC) has been presented for control of membrane potential of thalamic neuron populations through continuous adaptive DBS current applied to the thalamus. A fuzzy logic system (FLS) is used to estimate the unknown nonlinear dynamics of the model, and the weights of FLS are adjusted online to guarantee the convergence of FLS parameters to optimal values. The simulation results show that the proposed AFTSMC not only significantly produces lower tracking errors in comparison with the classical adaptive fuzzy sliding mode control (AFSMC), but also makes more robust and reliable outputs. The results suggest that the proposed AFTSMC provides a more robust and smooth control input which is highly desirable for hardware design and implementation.

## Introduction

Deep brain stimulation (DBS) has become an effective therapeutic solution for neurological disorders such as Parkinson’s disease (PD)^1–4^. In PD, degeneration of dopaminergic neurons leads to dopamine depletion in the substantia nigra pars compacta (SNc) which causes abnormal neuron activities. As a result, the thalamus (TH) relay reliability to sensorimotor commands is distorted^5–7^. DBS changes neural activities by delivering electrical currents to specific targets in the brain through implanted electrodes. The exact mechanisms underlying the DBS treatment are not clearly understood and are under debate^1,8^. In literature, the problem of DBS control for the treatment of PD can be approached at two mechanisms: open-loop^9–11^ and closed-loop^5,12–15^. Open-loop DBS involves high-frequency (around 130–180 Hz) trains of pulses with constant parameters without considering the state of disease. While open-loop DBS is efficient for alleviating PD symptoms, some technical challenges exist. The stimulation parameters are adjusted by a highly trained clinician expert to maximize the DBS efficiency and reducing its side effects^3^. Moreover, high-frequency stimulation consumes more energy which may reduce the battery longevity of the implanted device. In contrast, adaptive closed-loop DBS can be used to minimize stimulation-induced side effects by automatically determining the stimulation parameters based on the PD dynamics^12,13,16,17^, save energy, and reduce the risk of battery replacement in real DBS systems^2^. Furthermore, the feasibility of adaptive DBS has been shown previously using a fully implanted neural prosthesis^18^. During recent years various control approaches have been developed and tested on the computational models of PD^5,7,14,19–21^. Santaniello et al. developed a closed-loop control system using a recursively identified autoregressive model (ARX) to adjust the stimulation amplitude based on the feedback of electrical signals recorded from the brain^14^. A similar ARX structure was used as the predictive model in the generalized predictive control (GPC) method to generate the optimal stimulation pulses and modulate the activities of the neuronal basal ganglia (BG) model^5^. In^19^, a nonlinear predictive control scheme based on an ARX model has been addressed to online adjustment of DBS amplitude and frequency. Su et al. proposed an adaptive feedback linearization (FL) algorithm to restore thalamic neurons relay reliability^7^. The major disadvantage of the proposed FL approach is that to design the control law the dynamics of a highly nonlinear computational model of PD are required. In^20,21^, a simple closed-loop DBS based on linear delayed feedback has been suggested to effectively desynchronize a model of two neuronal populations of the subthalamic nucleus (STN) and the external segment of the globus pallidum (GPe). The aforementioned control works assumed that the dynamics of the model are known with unknown slow-varying system parameters. Moreover, these methods suffer from several issues, such as transient performance, unmodeled dynamics, the amount of offline training, and system stability issues in real applications with the external disturbances and uncertainties of the highly nonlinear computational model of PD being controlled.

Sliding mode control (SMC) is a robust and powerful control technique to handle the nonlinear uncertain system in the presence of bounded external disturbances^22–24^. Zhu et al. proposed a robust control technique based on SMC for control of membrane potential of a thalamic neuron in a thalamocortical computational model of BG network consisting of STN, GPe, internal segment of the globus pallidum (GPi), and TH^6^. The control objective was to design a DBS waveform to force the membrane potential of the thalamic neuron to track the normal firing pattern in real-time in a closed-loop manner using the feedback signal. The main limitation of the work is that to design the control pulses, the BG model dynamics should be assumed known. Moreover, the main drawback of the conventional SMC is that due to the linear switching of the manifolds the tracking error of the system converges to the origin asymptotically. To resolve the global asymptotic stability of conventional SMC, terminal sliding mode (TSM) control guarantees the finite-time convergence of the system states to the origin^24,25^. By using fractional-power terms instead of linear in switching surface of conventional SMC, the fast convergence of the TSM control in finite time is guaranteed. However, the main drawbacks of the discontinuous TSM are the singularity of the control input^26,27^ and chattering problems^25,28^. In the singularity condition, the amplitude of the control input in some areas of the state space may increase infinitely to guarantee the ideal TSM motion. In chattering phenomena, the high-frequency unmodeled dynamics of the system may be occurred due to the discontinuous switching of the control input across the sliding surface.

To resolve all the above problems, in this paper, we present a fast adaptive fuzzy terminal sliding mode control (AFTSMC) to control the membrane potential of thalamic neuron populations in a BG–thalamic network model. The proposed controller generates an adaptive control signal (stimulation current applied to the TH) automatically to force the firing patterns of the Parkinsonian state to track the normal firing patterns. The main innovations of the work are as follows:

- In^6^, the problem of robust control of a single thalamic neuron with uncertain external disturbance has been addressed. In contrast, in the current study, by modeling the interaction between the neurons in populations of the thalamic, the network-level modulation of thalamic is represented in a standard canonical form as the multi-input multi-output (MIMO) nonlinear first-order system with uncertainty and external disturbances.
- To increase the speed of the controller outside the sliding surface and eliminate the chattering problem, a fast continuous TSM-type reaching term is designed to ensure the finite-time motion of the system states to the sliding manifold.
- On the surface, a nonsingular continuous integral fractional-power surface is developed to ensure the bounded finite-time convergence of the tracking error.
- A fuzzy logic system (FLS) is used to estimate the unknown nonlinear dynamics of the model embedded in the control input, and the weights of FLS are adjusted online to guarantee the convergence of FLS parameters to optimal values.

Simulation results are given to evaluate the performance of the proposed control method through the control of firing patterns of Parkinsonian state to track the normal state of the TH, and the results are compared with the adaptive fuzzy conventional SMC. The simulation results show the effectiveness of the proposed AFTSMC in deal with the uncertainty and external disturbances of ionic channels.

## Model and control problem

### Model

**Figure 1:**
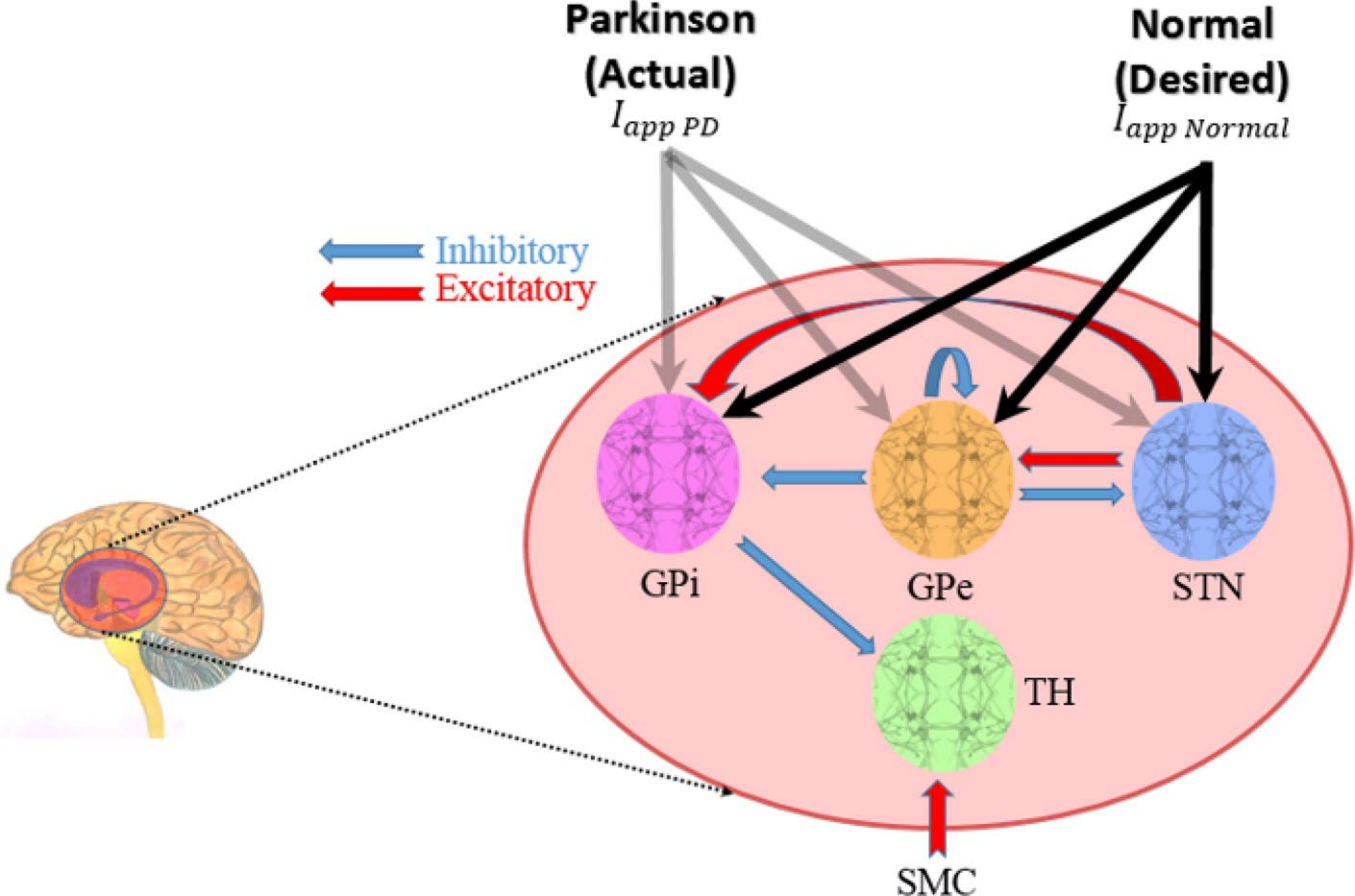
The BG-thalamic network layout for normal and PD conditions. Healthiness or Parkinsonian state is modeled by applying a specific set of *I*_*app*_ currents to GPi, GPe, and STN populations (grey and black arrows for Parkinson and healthy states, respectively). The interconnections between different neuronal populations are also depicted which shows different excitatory or inhibitory connections between neurons. Excitatory and inhibitory connections are shown by red and blue arrows, respectively. The sensorimotor cortex excitatory current to the TH is denoted by SMC.

The model of the BG-thalamic network simulated in this study is adopted from So et al*.*^11^. In this model, four neuronal populations (10 neurons for each population) are modeled for TH, STN, GPe, and GPi. The network topology and connections are shown in Fig. 1. For each neuron in GPe (GPi) population there exist two inhibitory inputs from GPe neurons and two excitatory inputs from STN neurons. Each GPi neuron inhibits a TH neuron, and each STN neuron is inhibited by two GPe neurons. Hence, the dynamic variations of the GPi population and its disturbances or uncertainties originated from the two populations GPe and STN, may directly or indirectly affect the TH. To model the state of Parkinson, the net bias current shown by *I*_*app*_ is reduced to show the dopamine depletion in SNc. The value of *I*_*app*_ for different populations at both healthy and PD conditions is provided in Table 1. The membrane potential dynamics of TH, STN, GPE, and GPi are modeled by the Hodgkin-Huxley (HH) equations as follows:

1 $$C_{m} \frac{{dv_{TH} }}{dt} = - I_{L} - I_{Na} - I_{K} - I_{T} - I_{GPi \to TH} + I_{SMC} + I_{dbs}$$

2 $$C_{m} \frac{{dv_{STN} }}{dt} = - I_{L} - I_{Na} - I_{K} - I_{T} - I_{Ca} - I_{ahp} - I_{GPe \to STN} + I_{app\_STN}$$

3 $$C_{m} \frac{{dv_{GPe} }}{dt} = - I_{L} - I_{Na} - I_{K} - I_{T} - I_{Ca} - I_{ahp} - I_{STN \to GPe} + I_{GPe\_GPe} + I_{app\_GPe}$$

4 $$C_{m} \frac{{dv_{GPi} }}{dt} = - I_{L} - I_{Na} - I_{K} - I_{T} - I_{Ca} - I_{ahp} - I_{STN \to GPi} + I_{GPe\_GPi} + I_{app\_GPi} ,$$

**Table 1:** The net bias applied currents (uA/cm^2^) to the different BG populations for the healthiness and PD states.

State	STN	GPe	GPi
Healthy	33	20	21
PD	23	7	15

where in (1)–(4),$v_{i} , \, i \in \left\{ {TH,STN,GPe,GPi} \right\}$ denotes the membrane potential of a single *STN, TH*, *GPe*, and *GPi* neuron respectively, $C_{m} = 1\mu Fcm^{ - 2}$ indicates the membrane capacitance.$I_{Na}$,$I_{K}$,$I_{Ca}$,$I_{T}$,$I_{ahp}$, and $I_{L}$ are the sodium current, potassium current, high-threshold calcium current, low-threshold calcium current, after hyperpolarization current, and leak current, respectively. The synaptic current between two neurons is denoted as follows:

5 $$I_{\alpha \to \beta } = g_{\alpha \to \beta } \left( {v_{\beta } - E_{\alpha \to \beta } } \right)\sum\limits_{j} {s_{j} } ,$$

where $\alpha$ and $\beta$ are representing pre and post-synaptic neurons, respectively. $g_{\alpha \to \beta }$ denotes the maximal synaptic conductance and $E_{\alpha \to \beta }$ denotes the reverse synaptic potential. The summation term over $s_{j}$ shows the presynaptic neurons current integration. The sensorimotor cortex (SMC) excitatory current to the TH is denoted by $I_{SMC}$ which is defined as follows:

6 $$I_{SMC} = i_{SMC} H\left( {\sin \left( {{{2\pi t} \mathord{\left/ {\vphantom {{2\pi t} {\rho_{SMC} }}} \right. \kern-\nulldelimiterspace} {\rho_{SMC} }}} \right)} \right) \times \left[ {1 - H\left( {\sin \left( {2\pi \left( {{{t + \delta_{SMC} } \mathord{\left/ {\vphantom {{t + \delta_{SMC} } {\rho_{SMC} }}} \right. \kern-\nulldelimiterspace} {\rho_{SMC} }}} \right)} \right)} \right)} \right],$$

where *H* is a Heaviside step function.$i_{SMC} = 3.5uAcm^{ - 2}$, ${1 \mathord{\left/ {\vphantom {1 {\rho_{SMC} }}} \right. \kern-\nulldelimiterspace} {\rho_{SMC} }}$, and $\delta_{SMC} = 5{\text{ ms}}$ are the amplitude, frequency, and duration of the pulse, respectively. Due to the non-regular nature of SMC input, the frequency of the pulse ${1 \mathord{\left/ {\vphantom {1 {\rho_{SMC} }}} \right. \kern-\nulldelimiterspace} {\rho_{SMC} }}$ is generated from a gamma distribution with an average rate of 14 Hz and a coefficient of variation of 0.2^19^. All the TH neurons in the population receive the SMC current with different initial voltages and the SMC pulse at each time forces them to fire. $I_{dbs}$ is the stimulation pulses delivered to the TH and designed in the control problem section. A detailed description of the model equations is provided in Appendix A of the Supplementary Materials.

Simulation of the system including the BG-network model and adaptive fuzzy MIMO controller uses MATLAB R2020a for implementing and obtaining the adaptive continuous DBS pulses. The entire period of simulation is 1000 ms (sampling period, 0.01 ms for control updates). The root mean square (RMS) of the absolute tracking error (*RMSE*) is calculated to measure the tracking accuracy as follows:

7 $$RMSE = \sqrt {\frac{1}{T}\sum\limits_{t = 1}^{T} {\left| {v_{TH} (t) - v_{THd} (t)} \right|^{2} } } ,$$

where $v_{TH}$ and $v_{THd}$ are the membrane potentials of each thalamic neuron for Parkinsonian and healthy states, respectively and *T* is the entire time of the simulation. Moreover, to quantify the performance of the stimulation pulses, the energy index is defined as the RMS of the control input in the following form^19^:

8 $$Energy = \sqrt {\frac{1}{T}\sum\limits_{t = 1}^{T} {{\mathbf{u}}^{T} (t){\mathbf{u}}(t)} } .$$

### Control problem

The BG–thalamic network model described in (1)–(4) is used as a PD model. To obtain the control input (DBS stimulation) using AFTSMC, the dynamics of thalamic neurons in (1) should be considered in the following canonical form:

9 $${\dot{\mathbf{x}}}(t) = {\mathbf{f}}_{1} ({\mathbf{x}},t) + {\mathbf{F}}_{2} ({\mathbf{x}},t) \cdot {\mathbf{u}}(t) + {\mathbf{d}}(t),$$

where ${\mathbf{x}} = [x_{1} , \ldots , \, x_{m} ]^{T}$ is a measurable state vector (membrane potentials of thalamic neurons) and ${\mathbf{u}} = [u_{1} , \ldots , u_{m} ]^{T}$ indicates the control input (DBS pulses).${\mathbf{d}}(t)$ denotes unknown bounded external perturbations and uncertainties of the system i.e., $\left\| {{\mathbf{d}}(t)} \right\| < \upsilon$, where $\upsilon$ is a nonnegative known value. The unknown vector functions ${\mathbf{f}}_{1} ({\mathbf{x}},t) + {\mathbf{d}}(t)$ and ${\mathbf{F}}_{2} ({\mathbf{x}},t)$ are defined as

10 $${\mathbf{f}}_{1} ({\mathbf{x}},t) = \left[ {f_{{{1}_{{1}} }} {(}{\mathbf{x}},t{),} \ldots {, }f_{{{1}_{{\text{m}}} }} {(}{\mathbf{x}},t{)}} \right]^{T} ,$$

11 $${\mathbf{F}}_{2} ({\mathbf{x}},t) = \left[ {\begin{array}{*{20}c} {f_{{2_{11} }} ({\mathbf{x}},t)} & \cdots & {f_{{2_{1m} }} ({\mathbf{x}},t)} \\ \vdots & \ddots & \vdots \\ {f_{{2_{m1} }} ({\mathbf{x}},t)} & \cdots & {f_{{2_{mm} }} ({\mathbf{x}},t)} \\ \end{array} } \right].$$

#### Assumption 1

${\mathbf{F}}_{2} ({\mathbf{x}},t)$ is a positive definite matrix and a real parameter $\sigma_{0} > 0$ is exists such that ${\mathbf{F}}_{2} ({\mathbf{x}},t{)} > \sigma_{0} {\mathbf{I}}_{m}$, where ${\mathbf{I}}_{m}$ is an $m \times m$ identity matrix.

#### Assumption 2

The desired trajectory $x_{{d_{i} }} (t){, }i = 1, \ldots , m$ is a known (membrane potentials of thalamic neurons in normal condition) continuous function which its first-order dynamics are exist for measurement.

If the tracking error of the PD model is considered as $e_{i} = x_{{d_{i} }} - x_{i}$, then, to implement AFTSMC the nonsingular continuous sliding surface is designed as follows:

12 $$s_{i} (t) = \int\limits_{0}^{t} {e_{i} (t)dt} + \sigma sig(e_{i} (t))^{\eta } ,\;\;\;i = 1,\ldots,m$$

where $sig(e_{i} (t))^{\eta } = \left| {e_{i} (t)} \right|^{\eta } sign(e_{i} (t))$, $\sigma$ and $\eta$ are positive design parameters that satisfy $\sigma > 0,1 < \eta < 2$. If the initial system states are away from the switching surface, a fast reaching law is designed to guarantee the finite-time motion of the system states to the sliding manifold as follows:

13 $${\dot{\mathbf{s}}} = - {\mathbf{K}}_{{\mathbf{1}}} sig({\mathbf{s}})^{{\rho_{1} }} - {\mathbf{K}}_{{\mathbf{2}}} sig({\mathbf{s}})^{{\rho_{2} }} ,$$

where the matrices ${\mathbf{K}}_{1} = diag(k_{11} ,\ldots,k_{1m} ) > {\mathbf{0}}_{m \times m}$ and ${\mathbf{K}}_{2} = diag(k_{21} ,\ldots,k_{2m} ) > {\mathbf{0}}_{m \times m}$ are design control gains and $1 < \rho_{1} < 3,0 < \rho_{2} < 1$. The first dynamic of the sliding surface vector is

14 $${\dot{\mathbf{s}}} = {\mathbf{e}} + \eta \sigma diag(\left| {\mathbf{e}} \right|^{\eta - 1} ){\dot{\mathbf{e}}}.$$

The equivalent control input is designed as follows:

15 $${\mathbf{u}}_{eq} (t) = {\mathbf{F}}_{2}^{ - 1} ({\mathbf{x}},t)\left( { - {\mathbf{f}}_{1} ({\mathbf{x}},t) - {\mathbf{d}}(t) + {\dot{\mathbf{x}}}_{d} (t) + \frac{1}{\eta \sigma }sig\left( {{\mathbf{e}}(t)} \right)^{2 - \eta } + {\mathbf{K}}_{1} sig({\mathbf{s}})^{{\rho_{1} }} + {\mathbf{K}}_{2} sig({\mathbf{s}})^{{\rho_{2} }} } \right)$$

#### Lemma 1

*If an extended Lyapunov function* $V({\mathbf{x}})$ *is given as follows*:

16 $$\dot{V}({\mathbf{x}}) + \alpha_{1} V^{{\lambda_{1} }} ({\mathbf{x}}) + \alpha_{2} V^{{\lambda_{2} }} ({\mathbf{x}}) \le 0,$$

where $\alpha_{1} ,\alpha_{2} > 0$ and $\lambda_{1} \ge 1,0 < \lambda_{2} < 1$, then, its settling time is given by

17 $$t_{s} \le \frac{{V({\mathbf{x}}_{0} )^{{1 - \lambda_{1} }} }}{{\alpha_{1} \left( {\lambda_{1} - 1} \right)}} \cdot F\left( {1,\frac{{\lambda_{1} - 1}}{{\lambda_{1} - \lambda_{2} }};\frac{{\lambda_{1} - 1}}{{\lambda_{1} - \lambda_{2} }} + 1; - \frac{{\alpha_{2} }}{{\alpha_{1} }}V({\mathbf{x}}_{0} )^{{\lambda_{2} - \lambda_{1} }} } \right),$$

where $F(a,b;c;z)$ indicates Gauss’s hypergeometric function^29^. The settling time of the Lemma 1 is proved in Appendix B of the Supplementary Materials.

#### Lemma 2

If $a_{1} ,a_{2} ,\ldots,a_{n}$ are all positive parameters, and $0 < p \le 2$, then the following inequality always maintains^30^:

18 $$\left( {a_{1}^{2} + \ldots + a_{n}^{2} } \right)^{p} \le \left( {a_{1}^{p} + \ldots + a_{n}^{p} } \right)^{2} .$$

#### Theorem 1

*The nonlinear MIMO model defined in* (9) *with its Assumptions* 1*and* 2*is considered. The terminal switching manifold and reaching law are chosen as* (12) *and* (13), *respectively, and the control input is defined by* (15). *If the system states are away from the switching manifold, the dynamics of the model converge to the switching manifold* $s_{i} = 0$ *in a finite time.*

The proof is given in Appendix C of the Supplementary Materials. When the states reached the sliding surface (${\mathbf{s}} = 0$), the dynamics of (4) has a globally finite-time stable attractor in $e_{i} = 0$^31^, so that the convergence time $t_{r}$ is finite with any condition $x_{i} (t_{{r_{i} }} )$ and is calculated as follows:

19 $$t_{{s_{i} }} = \frac{\sigma }{{1 - \frac{1}{\eta }}}\left| {x_{i} (t_{{r_{i} }} )} \right|^{\eta - 1} .$$

### Adaptive fuzzy terminal sliding mode control (AFTSMC)

In real applications of closed-loop DBS systems, the dynamics of functions ${\mathbf{f}}_{1} ({\mathbf{x}},t) + {\mathbf{d}}(t)$ and ${\mathbf{F}}_{2} ({\mathbf{x}},t)$ are unavailable and the control input (15) cannot exist. In the current study, to resolve the problem, FLS is applied to estimate these unknown dynamics (see details in Appendix D of the Supplementary Materials). If ${\hat{\mathbf{f}}}_{1} ({\mathbf{x}},{{\varvec{\uppsi}}}_{{f_{1} }}^{t} )$ and ${\hat{\mathbf{F}}}_{2} ({\mathbf{x}},{{\varvec{\uppsi}}}_{{f_{2} }}^{t} )$ are the fuzzy approximations of ${\mathbf{f}}_{1} ({\mathbf{x}},t) + {\mathbf{d}}(t)$ and ${\mathbf{F}}_{2} ({\mathbf{x}},t)$, respectively, the following modified control input (15) is written:

20 $$\begin{gathered} {\mathbf{u}}_{eq} (t) = \frac{{{\hat{\mathbf{F}}}_{2}^{T} ({\mathbf{x}},{{\varvec{\uppsi}}}_{{f_{2} }}^{t} )}}{{\varepsilon_{0} {\mathbf{I}}_{m} + {\hat{\mathbf{F}}}_{2} ({\mathbf{x}},{{\varvec{\uppsi}}}_{{f_{2} }}^{t} ){\hat{\mathbf{F}}}_{2}^{T} ({\mathbf{x}},{{\varvec{\uppsi}}}_{{f_{2} }}^{t} )}} \times \hfill \\ \, \left( { - {\hat{\mathbf{f}}}_{1} ({\mathbf{x}},{{\varvec{\uppsi}}}_{{f_{1} }}^{t} ) + {\dot{\mathbf{x}}}_{d} (t) + \frac{1}{\eta \sigma }sig({\mathbf{e}}(t))^{2 - \eta } + {\mathbf{K}}_{1} sig({\mathbf{s}})^{{\rho_{1} }} + {\mathbf{K}}_{2} sig({\mathbf{s}})^{{\rho_{2} }} } \right), \hfill \\ \end{gathered}$$

where $\varepsilon_{0}$ is a very small positive number. If the inverse of ${\hat{\mathbf{F}}}_{2} ({\mathbf{x}},{{\varvec{\uppsi}}}_{{f_{2} }}^{t} )$ cannot exist, the regularized form of ${\hat{\mathbf{F}}}_{2} ({\mathbf{x}},{{\varvec{\uppsi}}}_{{f_{2} }}^{t} )^{ - 1}$ in (18) is used. With the regularized definition ${\hat{\mathbf{F}}}_{2} ({\mathbf{x}},{{\varvec{\uppsi}}}_{{f_{2} }}^{t} )^{ - 1}$, the control signal (20) can always be well defined. Furthermore, a corrective control term is added to the main control input for compensating the effect of approximation errors in the following form:

21 $${\mathbf{u}}(t) = {\mathbf{u}}_{eq} (t) + {\mathbf{u}}_{c} (t),$$

where ${\mathbf{u}}_{eq} (t)$ is given in (18) and ${\mathbf{u}}_{c} (t)$ is proposed as follows:

22 $${\mathbf{u}}_{c} (t) = \frac{{{\mathbf{s}}\left| {{\mathbf{s}}^{T} } \right| \, ({\overline{\mathbf{\varepsilon }}}_{{f_{1} }} + \overline{\varepsilon }_{{f_{2} }} \left| {{\mathbf{u}}_{eq} } \right| + \left| {{\mathbf{u}}_{0} } \right|)}}{{\sigma_{0} \left\| {\mathbf{s}} \right\|^{2} + \Upsilon }}.$$

where $\sigma_{0} > 0,$ and $\Upsilon$ is an adjustable parameter designed with the following equation:

23 $$\dot{\Upsilon } = - \kappa_{0} \frac{{\left| {{\mathbf{s}}^{T} } \right|\eta \sigma diag(\left| {\mathbf{e}} \right|^{\eta - 1} )\left( {{\overline{\mathbf{\varepsilon }}}_{{f_{1} }} + \overline{\varepsilon }_{{f_{2} }} \left| {{\mathbf{u}}_{eq} } \right| + \left| {{\mathbf{u}}_{0} } \right|} \right)}}{{\sigma_{0} \left\| {\mathbf{s}} \right\|^{2} + \Upsilon }}.$$

In (22) and (23), ${\mathbf{u}}_{0} (t)$ is as follows:

24 $$\begin{gathered} {\mathbf{u}}_{0} (t) = \varepsilon_{0} (\varepsilon_{0} {\mathbf{I}}_{m} + {\hat{\mathbf{F}}}_{2} ({\mathbf{x}},{{\varvec{\uppsi}}}_{{f_{2} }}^{t} ){\hat{\mathbf{F}}}_{2}^{T} ({\mathbf{x}},{{\varvec{\uppsi}}}_{{f_{2} }}^{t} ))^{ - 1} \times \hfill \\ \, \left( { - {\hat{\mathbf{f}}}_{1} ({\mathbf{x}},{{\varvec{\uppsi}}}_{{f_{1} }}^{t} ) + {\dot{\mathbf{x}}}_{d} (t) + \frac{1}{\eta \sigma }sig({\mathbf{e}}(t))^{2 - \eta } + {\mathbf{K}}_{1} sig({\mathbf{s}})^{{\rho_{1} }} + {\mathbf{K}}_{2} sig({\mathbf{s}})^{{\rho_{2} }} } \right). \hfill \\ \end{gathered}$$

#### Theorem 2

*Consider the nonlinear model of the system* (9) *with nonlinear time-varying dynamics* ${\mathbf{f}}_{1} {\mathbf{(x}},t{\mathbf{)}}, \, {\mathbf{d(}}t{\mathbf{)}}$ *and* ${\mathbf{F}}_{2} {\mathbf{(x}},t{\mathbf{)}}$, *which are estimated with (S.23) and (S.24), and the Assumptions* 1 and 2*hold. The control signal is selected as* (21) *and the adaptive rules are considered as (S.30), (S.31), and* (23).* Thus, the following results are proven:*

1. *The parameter vectors* ${{\varvec{\uppsi}}}_{{f_{1} }}^{t}$ *and* ${{\varvec{\uppsi}}}_{{f_{2} }}^{t}$ *converge to* ${{\varvec{\uppsi}}}_{{f_{1} }}^{*}$ *and* ${{\varvec{\uppsi}}}_{{f_{2} }}^{*}$ *asymptotically.*
2. *If* ${\mathbf{f}}_{1}^{*} ({\mathbf{x}},{{\varvec{\uppsi}}}_{{f_{1} }}^{*} ) = {\hat{\mathbf{f}}}_{1} ({\mathbf{x}},{{\varvec{\uppsi}}}_{{f_{1} }}^{t} )$ *and* ${\mathbf{F}}_{2}^{*} ({\mathbf{x}},{{\varvec{\uppsi}}}_{{f_{2} }}^{*} ) = {\hat{\mathbf{F}}}_{2} ({\mathbf{x}},{{\varvec{\uppsi}}}_{{f_{2} }}^{t} )$*, then, the finite-time convergence of tracking error to the origin is guaranteed.*
3. *If* ${\mathbf{f}}_{1}^{*} ({\mathbf{x}},{{\varvec{\uppsi}}}_{{f_{1} }}^{*} ) \ne {\hat{\mathbf{f}}}_{1} ({\mathbf{x}},{{\varvec{\uppsi}}}_{{f_{1} }}^{t} )$ *and* ${\mathbf{F}}_{2}^{*} ({\mathbf{x}},{{\varvec{\uppsi}}}_{{f_{2} }}^{*} ) \ne {\hat{\mathbf{F}}}_{2} ({\mathbf{x}},{{\varvec{\uppsi}}}_{{f_{2} }}^{t} )$*, then, the sliding variable converges to the neighborhood of zero as follows:*
  25 $$\left\| {\mathbf{s}} \right\| \le \left( {\frac{{\left\| {{\hat{\mathbf{f}}}_{1} ({\mathbf{x}},{{\varvec{\uppsi}}}_{{f_{1} }}^{t} ) - {\mathbf{f}}_{1}^{*} ({\mathbf{x}},{{\varvec{\uppsi}}}_{{f_{1} }}^{*} )} \right\| + \left\| {{\hat{\mathbf{F}}}_{2} ({\mathbf{x}},{{\varvec{\uppsi}}}_{{f_{2} }}^{t} ) - {\mathbf{F}}_{2}^{*} ({\mathbf{x}},{{\varvec{\uppsi}}}_{{f_{2} }}^{*} )} \right\|\left\| {{\mathbf{u}}_{eq} } \right\|}}{{k_{1} }}} \right)^{{1/\rho_{1} }} = \delta_{1} ,$$
  26 $$\left\| {\mathbf{s}} \right\| \le \left( {\frac{{\left\| {{\hat{\mathbf{f}}}_{1} ({\mathbf{x}},{{\varvec{\uppsi}}}_{{f_{1} }}^{t} ) - {\mathbf{f}}_{1}^{*} ({\mathbf{x}},{{\varvec{\uppsi}}}_{{f_{1} }}^{*} )} \right\| + \left\| {{\hat{\mathbf{F}}}_{2} ({\mathbf{x}},{{\varvec{\uppsi}}}_{{f_{2} }}^{t} ) - {\mathbf{F}}_{2}^{*} ({\mathbf{x}},{{\varvec{\uppsi}}}_{{f_{2} }}^{*} )} \right\|\left\| {{\mathbf{u}}_{eq} } \right\|}}{{k_{2} }}} \right)^{{1/\rho_{2} }} = \delta_{2} ,$$

*where* $k_{1}$ *and* $k_{2}$ *denote the minimum eigenvalues of matrices* ${\mathbf{K}}_{1}$ *and* ${\mathbf{K}}_{2}$*, respectively. By virtue of * (25) *and* (26)*, the region* $\left\| {\mathbf{s}} \right\| \le \delta = \min (\delta_{1} ,\delta_{2} )$ *will be achieved in finite time. Then, the tracking error converges to a boundary layer*

27 $$\left| {e_{i} (t)} \right| \le \left( {\frac{\delta }{\sigma }} \right)^{{\frac{1}{\eta }}} , \, i = 1,\ldots,m$$

*in a finite time*.

The proof is given in Appendix E of the Supplementary Materials.

#### *Remark 1*

Due to the bounds of (25) and (26), the larger selection of control gains ${\mathbf{K}}_{1}$ and ${\mathbf{K}}_{2}$ results in a smaller boundary region $\delta$. But, by increasing the value of these parameters, the amplitude of the control signal (DBS pulses) will increase so that the control input may not be implemented.

#### *Remark 2*

Based on the results of Theorem 2, (25), and (26), the region $\delta$ converges to zero asymptotically.

#### *Remark 3*

The terms $sig({\mathbf{s}})^{{\rho_{1} }}$ and $sig({\mathbf{s}})^{{\rho_{2} }}$ in control law, and $\eta$ in the sliding surface are considered as a bridge between classical adaptive fuzzy sliding mode control ($\rho_{1} \to 1,\rho_{2} \to 0,\eta \to 1$) and AFTSMC ($1 < \rho_{1} < 3,0 < \rho_{2} < 1,1 < \eta < 2$). These parameters should be adjusted appropriately to guarantee to reach the sliding manifold in finite time and continuous control input.

## Simulation results

**Figure 2:**
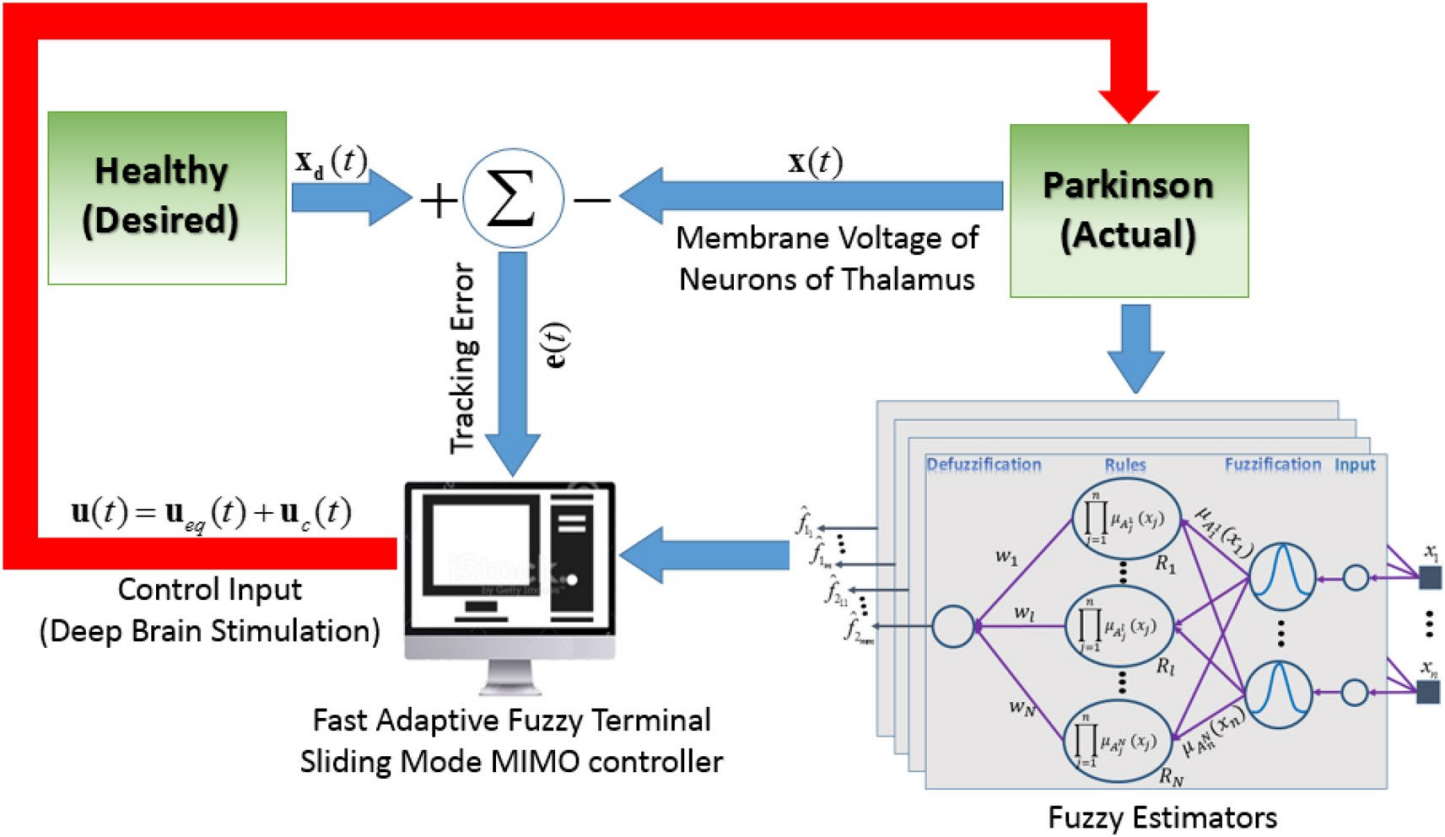
Closed-loop diagram of the proposed robust AFTSMC for control of Parkinsonian state through adaptive continuous DBS pulses. Tracking error,${\mathbf{e}}(t)$, is achieved by comparing the output of the healthy model, ${\mathbf{x}}_{d} (t)$, and the actual model, ${\mathbf{x}}(t)$. The fuzzy estimators receive the voltages of TH neurons as inputs and calculate the elements of ${\mathbf{f}}_{1}$ vector and ${\mathbf{F}}_{2}$ matrix as main parts of the nonlinear dynamic model of the TH (see details in Appendix D of the Supplementary Materials). ${\mathbf{f}}_{1}$, ${\mathbf{F}}_{2}$ and tracking error ${\mathbf{e}}(t)$ are used to compute the final control input ${\mathbf{u}}(t)$.

In this section, the results of the proposed AFTSMC to control the membrane potentials of the TH in Parkinsonian state are reported and evaluated. The closed-loop diagram of the proposed robust AFTSMC for control of Parkinsonian state is illustrated in Fig. 2. The control input is a continuous adaptive DBS current applied to the TH so that a DBS waveform forces the membrane potential of the thalamic neuron to track the normal firing pattern in real-time in a closed-loop manner using the feedback signal.

**Figure 3:**
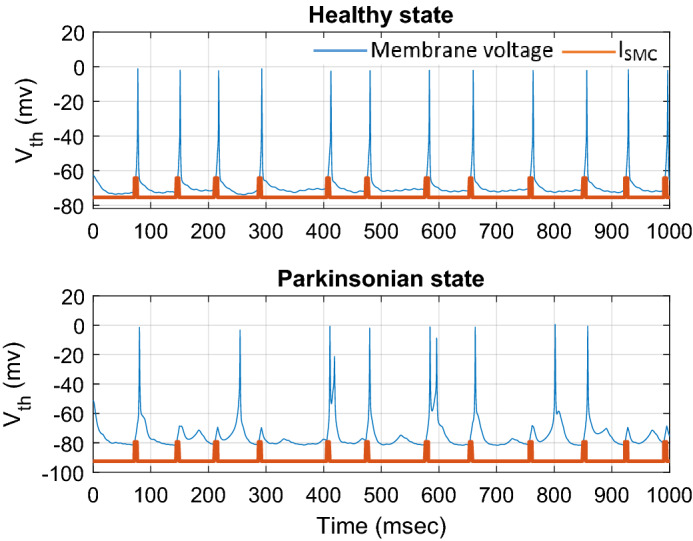
Results of the thalamic firing pattern for neuron 1 in response to the sensorimotor cortex (SMC) excitatory current to the TH for healthy and Parkinsonian states with the different net bias applied currents of Table 1 to the populations of *STN*, *GPe*, and *GPi*.

The fuzzy systems used to estimate ${\hat{\mathbf{f}}}_{1} ({\mathbf{x}},{{\varvec{\uppsi}}}_{{f_{1} }}^{t} )$ and ${\hat{\mathbf{F}}}_{2} ({\mathbf{x}},{{\varvec{\uppsi}}}_{{f_{2} }}^{t} )$ have the membrane potentials of thalamic neurons as input. For each state variable ${\mathbf{x}} = \left[ {v_{{TH_{1} }} , \ldots ,v_{{TH_{10} }} } \right]^{T}$ two Gaussian-type membership functions were defined as follows:

28 $$\mu_{{A_{j}^{i} }} = e^{{ - \frac{1}{2}\left( {\frac{{x_{j} - c_{j}^{i} }}{{\delta_{j} }}} \right)^{2} }} ,\;\;\;i = 1,2 \, j = 1,\ldots,10$$

where $c_{1}^{1} = c_{2}^{1} = \ldots = c_{10}^{1} = - 49.59,c_{1}^{2} = c_{2}^{2} = \ldots = c_{10}^{2} = - 25.36$ and $\delta_{1} = \delta_{2} = \ldots = \delta_{10} = 5$. The values of the adaptive FLS estimator are selected for covering the full possible range ${\mathbf{x}}$. The initial conditions of the BG-network states are random values between -60 and -70 mv, and the initial values of the parameter $\zeta$ are set to random values with a uniform distribution between 0 and 1. Figure 3 shows the results of the thalamic firing pattern for neuron 1 in response to SMC excitatory current to the TH for healthy and Parkinsonian. In the healthy state, as the SMC excitatory current pulses applied with a gamma distribution with an average rate of 14 Hz, the thalamic neuron responds to the input successfully, while in Parkinsonian state, due to the reduction of net bias applied currents to the populations of *STN*, *GPe*, and *GPi*, the spiking behavior of the neuron in the presence of the applied input current may be suppressed and the neuron failed to respond normally. The response of the TH neuron in the healthy state is considered as the desired pattern and the main objective is to design and generate the control input (adaptive continuous DBS pulses) automatically to force the firing patterns of the Parkinsonian state to track the normal firing patterns.

### Robust closed-loop control of DBS pulses

**Figure 4:**
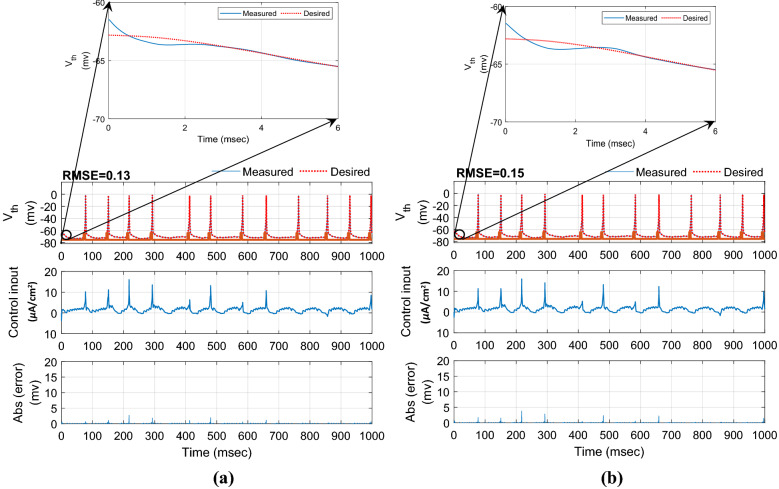
Typical results of the closed-loop control for neuron 1 using (**a**) AFTSMC (**b**) AFSMC.

**Figure 5:**
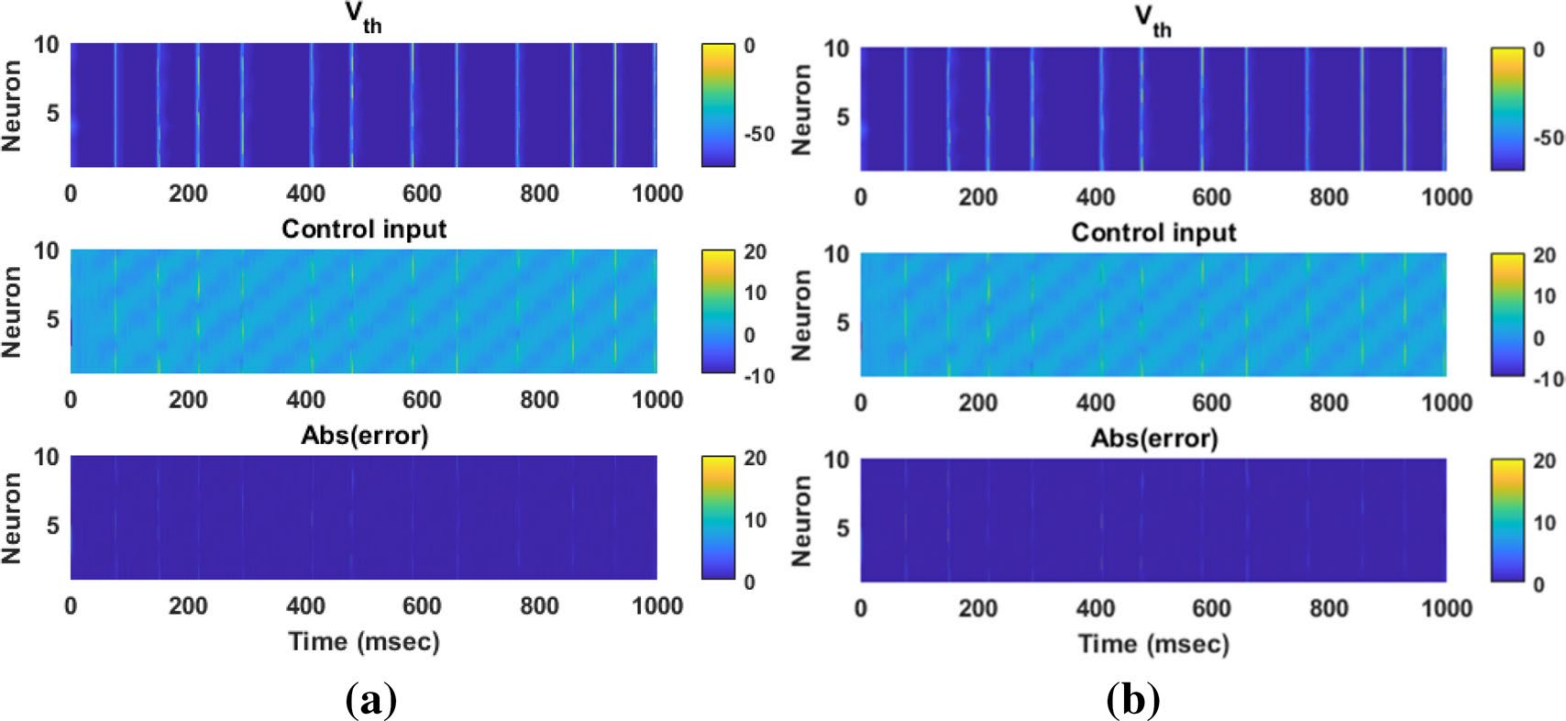
Typical results of the membrane potential, control input, and absolute tracking error for individual cells of the TH populations using (**a**) AFTSMC (**b**) AFSMC.

In this section, the results of controlling the firing patterns of Parkinsonian state through the proposed AFTSMC are evaluated. The control parameters (i.e., $\sigma ,\eta ,\rho_{1} ,\rho_{2} ,\varepsilon_{0} ,\kappa_{{f_{1} }} ,\kappa_{{f_{2} }} ,\kappa_{0} ,\sigma_{0} ,\Upsilon$) are selected with the trial-and-error process to reach the minimum tracking error with high accuracy and kept fixed during external disturbances and time-varying uncertainty simulations. Figure 4 shows the results of the tracking for neuron 1 using the proposed AFTSMC (Fig. 4a) in comparison with the classical adaptive fuzzy sliding mode control (AFSMC). The results show that *RMSE* is 0.13 mv and 0.15 mv for AFTSMC and classical AFSMC, respectively. The controller rapidly and automatically adjusted the level of stimulation signals to track the desired trajectory with the fast convergence speed. The membrane potential converges to the desired trajectory before 4 ms approximately. Figure 5 shows the membrane potential, control input, and absolute tracking error for individual cells of the TH populations. The bright bars in the chart of the membrane potential show the spiking activity of the thalamic cells in response to the input current of the sensorimotor cortex. The mean *RMSE* for 10 neurons of the TH is 0.15 mv and 0.19 mv for AFTSMC and classical AFSMC, respectively.

### Effects of system uncertainty (parameters variations)

**Figure 6:**
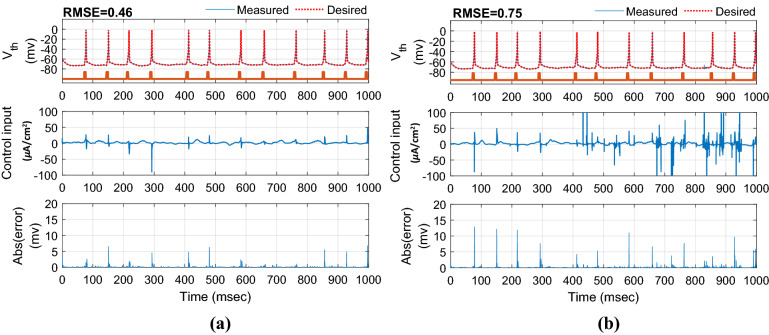
Typical results of the closed-loop control for neuron 1 under 50% time-varying system parameters using (**a**) AFTSMC (**b**) AFSMC.

**Figure 7:**
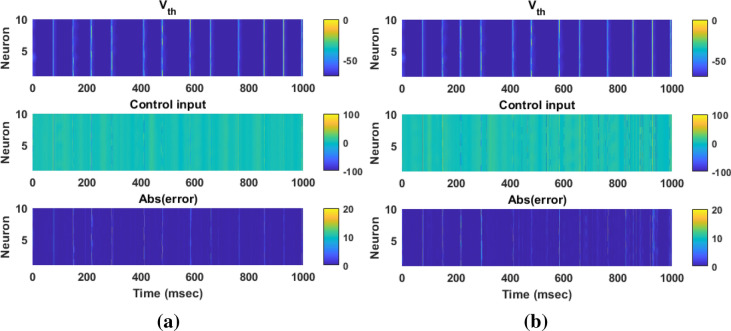
Typical results of the membrane potential, control input, and absolute tracking error for individual cells of the TH populations under 50% time-varying system parameters using (**a**) AFTSMC (**b**) AFSMC.

**Figure 8:**
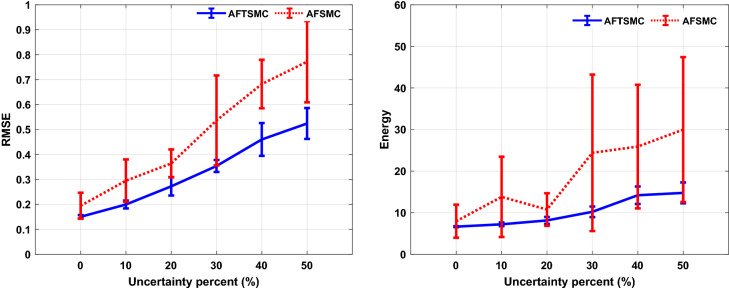
Results of the mean RMSE and Energy over 10 trials of the simulation under time-varying system parameters using AFTSMC in comparison with classical AFSMC. Standard deviation bars are shown for 10 independent simulations in each uncertainty.

In this section, to evaluate the performance of the proposed controller to handle time-varying uncertainty, the values of the system parameters were varied randomly about their nominal values during 1000 ms simulation. All model parameters which are presented in the supplementary tables are considered in this analysis. These parameters include maximal ionic conductance ($g_{L} ,g_{Na} ,g_{K} ,g_{T} ,g_{Ca} ,g_{ahp}$), reverse ionic potential ($E_{L} ,E_{Na} ,E_{K} ,E_{T} ,E_{Ca}$), maximum synaptic conductance ($g_{STN \to GPe} ,g_{STN \to GPi} ,g_{GPe \to STN} ,g_{GPe \to GPe} ,g_{GPe \to GPi} ,g_{GPi \to TH}$), and reverse synaptic potentials ($E_{STN \to GPe} ,E_{STN \to GPi} ,E_{GPe \to STN} ,E_{GPe \to GPe} ,E_{GPe \to GPi} ,E_{GPi \to TH}$). The variations were randomly acquired with the uniform distribution by passing the random sequences to the low-pass filter (fourth-order Butterworth-type) with the cutoff frequency of 0.025 Hz. Figure 6 shows the results of the tracking under 50% time-varying uncertainty of the parameters. The control input of AFTSMC is continuous without any chattering and singularity (*RMSE*, 0.46 mv). In contrast, due to the discontinuity of the classical AFSMC (*RMSE*, 0.75 mv) across the sliding surface, the control input generates high switching control activity which may excite unmodeled neglected dynamics of the BG-network, thus causing chattering. Figure 7 shows the membrane potential, control input, and absolute tracking error for individual cells of the TH populations. The averages of *RMSE* and *Energy* using AFTSMC and AFSMC over 10 trials of the simulation as a function of parameter variations from 0 to 50% are depicted in Fig. 8. Increasing the upper bound of the uncertainty caused more consumption of *Energy* and larger tracking error. The standard deviation (SD) and mean of *RMSE* and *Energy* generated using the AFSMC method were higher than that by the proposed AFTSMC. This indicates that the proposed AFTSMC method can provide robust control of DBS pulses with respect to the AFSMC. The results of the one-way ANOVA test show that the tracking performance and energy of the controllers AFTSMC and AFSMC were significantly different (*p* < 0.01).

### Effects of external disturbances and uncertainty

To evaluate the performance of the proposed AFTSMC to reject the external disturbance in the presence of uncertainty, the following external current is added to the dynamics of (1) for each TH neuron as the ionic channels disturbances:

29 $$i_{d} (t) = \sin \left( {1 + \frac{kt}{{10}}} \right),\;\;\;k = 1,\ldots,10.$$

**Figure 9:**
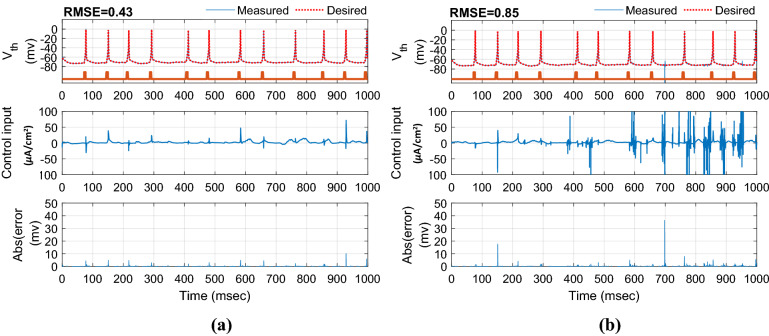
Typical results of the closed-loop control for neuron 1 under 50% time-varying system parameters and external disturbances using (**a**) AFTSMC (**b**) AFSMC.

**Figure 10:**
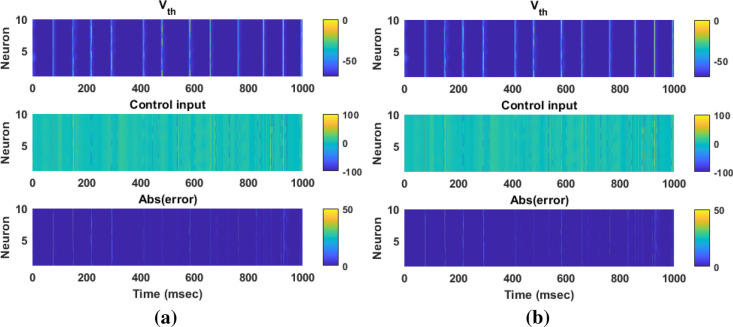
Typical results of the membrane potential, control input, and absolute tracking error for individual cells of the TH populations under 50% time-varying system parameters and external disturbances using (**a**) AFTSMC (**b**) AFSMC.

**Figure 11:**
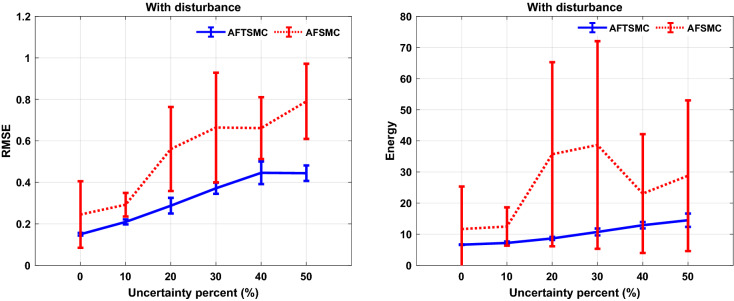
Results of the mean RMSE and Energy over 10 trials of the simulation under time-varying system parameters and external disturbance using AFTSMC in comparison with classical AFSMC. SD bars are depicted for 10 trials of simulations in each uncertainty.

Figure 9 shows the results of the tracking for neuron 1 using the proposed AFTSMC in comparison with the classical AFSMC. The results show that *RMSE* is 0.43 mv and 0.85 mv for AFTSMC and classical AFSMC, respectively. Figure 10 shows the membrane potential, control input, and absolute tracking error during the entire period of simulation for individual cells of the TH populations. The averages of *RMSE* and *Energy* using AFTSMC and AFSMC over 10 trials of the simulation as a function of parameter variations from 0 to 50% in the presence of external disturbance are indicated in Fig. 11. The parameters SD and mean of *RMSE* and *Energy* generated using the AFSMC method were higher than that by the proposed AFTSMC. The results of the one-way analysis of variance (ANOVA) test show that the tracking performance and energy of the controllers AFTSMC and AFSMC were significantly different (*p* < 0.01).

## Discussion and conclusions

In the current paper, a class of fast and robust MIMO control scheme based on AFTSMC has been presented for control of membrane potential of thalamic neuron populations in the BG–thalamic network model through continuous adaptive DBS pulses. In the previous work^6^, a robust SMC was proposed for modulation of Parkinsonian state with uncertain disturbance. A property of the classical SMC is the convergence of the tracking error to the origin in an infinite time. Moreover, in the previous work^6^, to design the control input the membrane potential of the thalamic neuron being controlled should be assumed known. In the current study, to resolve the limitation of globally asymptotic stabilization of classical SMC and speed up the convergence time in both reaching and sliding phases of the motion, an AFTSMC control has been proposed to guarantee the global stability of the closed-loop system with the fast speed. Another problem is the modeling of the complexity of the neuron populations in TH. From a simulation point of view, in^6^, a single-input single-output (SISO) framework was developed for robust control of a single thalamic neuron with uncertain external disturbance. In contrast, in this paper, the interaction between 10 neurons of the TH is represented as the MIMO nonlinear dynamic system and a class of MIMO control framework (centralized structure) is developed for controlling the BG system. Centralized controller structure requires a complex mathematical model of the BG dynamics in the control law designation and calculation. But, in real applications of robust closed-loop DBS systems, the challenge is the reduction of computational order of the system and easy implementation of the closed-loop system. One solution to cope with this limitation is the decentralized design of the system. In a decentralized control scheme, a system is divided into a set of subsystems in which an independent controller is used to control each subsystem. The controller of each subsystem is designed based on feedback measurements of the membrane potential of each isolated neuron of the TH. The external disturbance of each subsystem is considered as a model of the interaction between the subsystems and estimated online with the FLS. The real implementation of the proposed closed-loop controller in decentralized and centralized forms to evaluate the performance and real-time speed of each structure in PD patients through robust adaptive control of DBS using AFTSMC is considered as future research.

While computational modeling and simulation provide affordable tools to test and develop effective approaches before clinical trials, many methodological assumptions based on these computational models may not hold in real practical applications. Uncertainty and external disturbance are unavoidable elements of the brain neural networks, including ion channels and synaptic connections^32^. Furthermore, other sources of uncertainty, such as day-to-day and subject-to-subject variability, or possible inaccurate electrode targeting may increase the mismatch between the real control platform and the simulation-based designed controller. Hence, the control approaches purely depending on an accurate BG model, such as the feedback linearization method employed in^7^, would not be an ideal option for practical implementation. To take into account this issue, we evaluated the effects of uncertainty and disturbances on the performance of the proposed controller in a well quantitative manner by increasing the level of uncertainty from 0 to 50 percent with and without external disturbance circumstances. Although increasing uncertainty and/or disturbance negatively affected the control performance of both AFSMC and AFTSMC, the proposed AFTSMC significantly outperforms the classic AFSMC and its superior performance was statistically demonstrated. The proposed AFTSMC not only produced lower tracking errors but also made more robust and reliable outputs. Based on these results, the robustness against model uncertainty and disturbance can be considered as the most significant achievement of this study. This stability makes the proposed AFTSMC a reliable option for implementing in real applications.

In the current study, from a simulation point of view, a robust continuous adaptive control input (DBS current to the TH) could efficiently force the membrane potential of TH neuron populations to track the pattern of healthy subjects in Parkinsonian state without considering the frequency and parameters of the stimulation (pulse amplitude (PA) and pulse width (PW))^6,7^. In a real DBS platform, it can be seen that the stimulator can generate charge-balanced, biphasic current pulses^20,33,34^ with interphase delay, and both PA and PW of stimulation signal are individually or simultaneously adjusted by the AFTSMC to track the healthy patterns of the TH. The proposed controller has the ability of online adaptation to handle the subject and day-to-day variations or the environment changes. Another challenge in a real DBS platform is continuous stimulation with constant frequency. It may lead to increase side effects and battery usage. The optimal control strategy is of interest for current DBS technology to improve the energy efficiency for increasing the battery lifetime^35,36^. We considered this practical issue in our simulation by evaluating the energy index. The results demonstrated that under uncertainty and/or disturbance circumstances, the energy required for classic AFSMC will be highly variable, while the proposed AFTSMC energy index showed very stable values. Further considering the practical perspective, the control input resulted from classic AFSMC showed high switching activity, which makes it hard to achieve energy efficiency^37^. In contrast, the proposed AFTSMC provides a more robust and smooth control input which is highly desirable for hardware design and implementation.

## Supplementary Information

Supplementary Information.
