# Supplementary material for: Robust multi-input multi-output adaptive fuzzy terminal sliding mode control of deep brain stimulation in Parkinson’s disease: a simulation study
Source: Sci Rep. 2021 Oct 27;11:21169. doi: 10.1038/s41598-021-00365-9 (PMC8551209; doi:10.1038/s41598-021-00365-9)
Supplement: Supplementary file 1 — Supplementary Information. [file 41598_2021_365_MOESM1_ESM.docx]

**Supplementary Materials for**

**“Robust Multi-Input Multi-Output Adaptive Fuzzy Terminal Sliding Mode Control of Deep Brain Stimulation in Parkinson’s Disease: A Simulation Study”**

Ehsan Rouhani^1,*^ & Yaser Fathi^2^

^1^ Department of Electrical and Computer Engineering, Isfahan University of Technology, Isfahan 84156-83111, Iran. (Email: [erouhani@iut.ac.ir](mailto:erouhani@iut.ac.ir))

^2^ Department of Biomedical Engineering, School of Electrical Engineering, Iran University of Science and Technology, Tehran, Iran.

**Appendix A. Details of the model**

In this Appendix, the details of BG model equations and parameters are provided. The dynamic equations of ionic channels for the TH neurons are as follows:

$\frac{dh}{dt}=\left[h_{\infty}(v)-h\right]/\tau_{h}(v)$ (S.1)

$\frac{dr}{dt}=\left[r_{\infty}(v)-r\right]/\tau_{r}(v)$, (S.2)

where *h* and *r* are gating variables. The current equations and model parameters for TH neurons are summarized in Table S1.

**Table S1.** Current equations and model parameters for TH neurons.

| Current | Equation | Gating variables | Parameters |
| --- | --- | --- | --- |
| $I_{L}$ | $g_{L}\left(v-E_{L}\right)$ | $m_{\infty}(v)=1/\left(1+\exp\left(-\frac{v+37}{7}\right)\right)$ $p_{\infty}(v)=1/\left(1+\exp\left(-\frac{v+60}{6.2}\right)\right)$ $h_{\infty}(v)=1/\left(1+\exp\left(\frac{v+41}{4}\right)\right)$ $\tau_{h}(v)=1/\left(0.128\exp\left(-\frac{v+46}{18}\right)+4/\left(1+\exp\left(-\frac{v+23}{5}\right)\right)\right)$ $r_{\infty}(v)=1/\left(1+\exp\left(\frac{v+84}{4}\right)\right)$ $\tau_{r}(v)=0.15\left(28+\exp\left(-\frac{v+25}{10.5}\right)\right)$         | $g_{L}=0.05$ $E_{L}=-70$ $g_{Na}=3$ $E_{Na}=50$ $g_{K}=5$ $E_{K}=-75$ $g_{T}=5$ $E_{T}=0$   |
| $I_{Na}$ | $g_{Na}m_{\infty}(v)^{3}h\left(v-E_{Na}\right)$ |  |  |
| $I_{K}$ | $g_{K}\left[0.75\left(1-h\right)\right]\left(v-E_{K}\right)$ |  |  |
| $I_{T}$ | $g_{T}p_{\infty}(v)^{2}r\left(v-E_{T}\right)$ |  |  |

The dynamic equations of ionic channels for the subthalamic neurons (STNs) are as follows:

$\frac{dh}{dt}=0.75\left[h_{\infty}(v)-h\right]/\tau_{h}(v)$ (S.3)

$\frac{dn}{dt}=0.75\left[n_{\infty}(v)-n\right]/\tau_{n}(v)$ (S.4)

$\frac{dr}{dt}=0.2\left[r_{\infty}(v)-r\right]/\tau_{r}(v)$ (S.5)

$\frac{dc}{dt}=0.08\left[c_{\infty}(v)-c\right]/\tau_{c}(v)$ (S.6)

$\frac{d\left[Ca\right]}{dt}=3.75 \times10^{-5}\left(-I_{Ca}-I_{T}-22.5 \times\left[Ca\right]\right)$, (S.7)

where *n*, *h*, *c*, and *r* are gating variables and $\left[Ca\right]$ is the intracellular concentration of Ca^2+^. The current equations and model parameters for STNs are summarized in Table S2.

**Table S2.** Current equations and model parameters for STNs.

| Current | Equation | Gating variables | Parameters |
| --- | --- | --- | --- |
| $I_{L}$ | $g_{L}\left(v-E_{L}\right)$ | $m_{\infty}(v)=1/\left(1+\exp\left(-\frac{v+30}{15}\right)\right)$ $n_{\infty}(v)=1/\left(1+\exp\left(-\frac{v+32}{8}\right)\right)$ $\tau_{n}(v)=1+100/\left(1+\exp\left(-\frac{v+80}{-26}\right)\right)$ $a_{\infty}(v)=1/\left(1+\exp\left(-\frac{v+63}{7.8}\right)\right)$ $b_{\infty}(v)=1/\left(1+\exp\left(-\frac{r-0.4}{0.1}\right)\right)-1/\left(1+\exp(4)\right)$ $c_{\infty}(v)=1/\left(1+\exp\left(-\frac{v+20}{8}\right)\right)$ $\tau_{c}(v)=1+10/\left(1+\exp\left(\frac{v+80}{26}\right)\right)$ $h_{\infty}(v)=1/\left(1+\exp\left(\frac{v+39}{3.1}\right)\right)$ $\tau_{h}(v)=1+500/\left(1+\exp\left(-\frac{v+57}{-3}\right)\right)$ $r_{\infty}(v)=1/\left(1+\exp\left(\frac{v+67}{2}\right)\right)$ $\tau_{r}(v)=7.1+17.5/\left(1+\exp\left(-\frac{v+68}{-2.2}\right)\right)$                   | $g_{L}=2.25$ $E_{L}=-60$ $g_{Na}=37$ $E_{Na}=55$ $g_{K}=45$ $E_{K}=-80$ $g_{T}=0.5$ $E_{T}=0$ $g_{Ca}=2$ $E_{Ca}=140$ $g_{ahp}=20$ $E_{ahp}=-80$                       |
| $I_{Na}$ | $g_{Na}m_{\infty}(v)^{3}h\left(v-E_{Na}\right)$ |  |  |
| $I_{K}$ | $g_{K}n^{4}\left(v-E_{K}\right)$ |  |  |
| $I_{T}$ | $g_{T}a_{\infty}(v)^{3}b_{\infty}(r)^{2}r\left(v-E_{T}\right)$ |  |  |
| $I_{Ca}$ | $g_{Ca}c^{2}\left(v-E_{Ca}\right)$ |  |  |
| $I_{ahp}$ | $g_{ahp}\left(v-E_{ahp}\right)\left(\frac{\left[Ca\right]}{\left[Ca\right]+15}\right)$ |  |  |

The dynamic equations of ionic channels for the external and internal segments of globus pallidum (GPe and GPi) are as follows:

$\frac{dh}{dt}=0.75\left[h_{\infty}(v)-h\right]/\tau_{h}(v)$ (S.8)

$\frac{dn}{dt}=0.75\left[n_{\infty}(v)-n\right]/\tau_{n}(v)$ (S.9)

$\frac{dr}{dt}=0.2\left[r_{\infty}(v)-r\right]/30$ (S.10)

$\frac{d\left[Ca\right]}{dt}=1 \times10^{-4}\left(-I_{Ca}-I_{T}-15 \times\left[Ca\right]\right)$, (S.11)

where *n*, *h,* and *r* are gating variables and $\left[Ca\right]$ is the intracellular concentration of Ca^2+^. The current equations and model parameters for GPi (GPe) neurons are summarized in Table S3.

**Table S3.** Current equations and model parameters for GPi (GPe) neurons.

| Current | Equation | Gating variables | Parameters |
| --- | --- | --- | --- |
| $I_{L}$ | $g_{L}\left(v-E_{L}\right)$ | $m_{\infty}(v)=1/\left(1+\exp\left(-\frac{v+37}{10}\right)\right)$ $n_{\infty}(v)=1/\left(1+\exp\left(-\frac{v+50}{14}\right)\right)$ $\tau_{n}(v)=0.05+0.27/\left(1+\exp\left(-\frac{v+40}{-12}\right)\right)$ $a_{\infty}(v)=1/\left(1+\exp\left(-\frac{v+57}{2}\right)\right)$ $s_{\infty}(v)=1/\left(1+\exp\left(-\frac{v+35}{2}\right)\right)$ $h_{\infty}(v)=1/\left(1+\exp\left(\frac{v+58}{12}\right)\right)$ $\tau_{h}(v)=0.05+27/\left(1+\exp\left(-\frac{v+40}{-12}\right)\right)$ $r_{\infty}(v)=1/\left(1+\exp\left(\frac{v+70}{2}\right)\right)$             | $g_{L}=0.1$ $E_{L}=-65$ $g_{Na}=120$ $E_{Na}=55$ $g_{K}=30$ $E_{K}=-80$ $g_{T}=0.5$ $E_{T}=0$ $g_{Ca}=0.15$ $E_{Ca}=120$ $g_{ahp}=10$ $E_{ahp}=-80$                       |
| $I_{Na}$ | $g_{Na}m_{\infty}(v)^{3}h\left(v-E_{Na}\right)$ |  |  |
| $I_{K}$ | $g_{K}n^{4}\left(v-E_{K}\right)$ |  |  |
| $I_{T}$ | $g_{T}a_{\infty}(v)^{3}r\left(v-E_{T}\right)$ |  |  |
| $I_{Ca}$ | $g_{Ca}s_{\infty}(v)^{3}\left(v-E_{Ca}\right)$ |  |  |
| $I_{ahp}$ | $g_{ahp}\left(v-E_{ahp}\right)\left(\frac{\left[Ca\right]}{\left[Ca\right]+10}\right)$ |  |  |

The dynamics of synaptic currents ($I_{\alpha\to\beta}=g_{\alpha\to\beta}s_{\alpha}\left(v_{\beta}-E_{\alpha\to\beta}\right)$) are as follows:

For $STN\to GPe,\text{ }STN\to GPi,\text{ }GPi\to TH$

$\frac{ds}{dt}=z$ (S.12)

$\frac{dz}{dt}=0.234u(t)-0.4z-0.04s$. (S.13)

$u(t)=\left\{\begin{matrix} 1 \text{ } \text{if} \text{the} \text{presynaptic} \text{cell} \text{crosses} \text{a} \text{threshold of} -10 \text{mv} \text{at} \text{time} t \\ 0 \text{ } \text{Otherwise} \end{matrix}\right.$ (S.14)

For $GPe\to STN,\text{ }GPe\to GPi,\text{ }GPe\to GPe$

$\frac{ds}{dt}=2(1-s)H_{\infty}(v-20)-0.04s$ (S.15)

$H_{\infty}(v)=1/\left(1+\exp\left(-\frac{v+57}{2}\right)\right)$. (S.16)

**Table S4.** Parameters for synaptic currents.

| Parameter | Value | Parameter | Value |
| --- | --- | --- | --- |
| $g_{STN\to GPe}$ | 0.15 | $E_{STN\to GPe}$ | 0 |
| $g_{STN\to GPi}$ | 0.15 | $E_{STN\to GPi}$ | 0 |
| $g_{GPe\to STN}$ | 0.5 | $E_{GPe\to STN}$ | -85 |
| $g_{GPe\to GPe}$ | 0.5 | $E_{GPe\to GPe}$ | -85 |
| $g_{GPe\to GPi}$ | 0.5 | $E_{GPe\to GPi}$ | -85 |
| $g_{GPi\to TH}$ | 0.17 | $E_{GPi\to TH}$ | -85 |

**Appendix B. Proof of Lemma 1**

The Lyapunov function$V(\mathbf{x})$ has an equilibrium point $V=0$. As condition $0<\lambda_{2}<1$ holds, one may write and, therefore, the equilibrium point is an attractor of the infinite stability and the relaxation time for a solution with the initial condition $V=V(\mathbf{x}_{0})$ to this attractor is finite. By integrating both sides from zero to the settling time $t_{s}$, one may obtain:

$\begin{matrix} t_{s}\leq\int_{V(\mathbf{x}_{0})}^{0} \frac{-dV}{\alpha_{1}V^{\lambda_{1}}(\mathbf{x})+\alpha_{2}V^{\lambda_{2}}(\mathbf{x})} \\ \\ t_{s}\leq\frac{V(\mathbf{x}_{0})^{1-\lambda_{1}}}{\alpha_{1}\left(\lambda_{1}-1\right)}\cdot F\left(1,\frac{\lambda_{1}-1}{\lambda_{1}-\lambda_{2}};\frac{\lambda_{1}-1}{\lambda_{1}-\lambda_{2}}+1;-\frac{\alpha_{2}}{\alpha_{1}}V(\mathbf{x}_{0})^{\lambda_{2}-\lambda_{1}}\right). \end{matrix}$ (S.17)

This completes the proof of Lemma 1.

**Appendix C. Proof of Theorem 1**

If and using the MIMO dynamic equations of the system, substituting (15) into the first dynamics of the sliding surface (12) in the form of vector, one can obtain

(S.18)

By considering the Lyapunov function $V_{1}=0.5\mathbf{s}^{T}\mathbf{s}$ and differentiating it with respect to the time, and substituting (S.18), we have:

, (S.19)

where $\bar{\mathbf{K}}_{1}=\eta\sigma diag(\left|\mathbf{e}\right|^{\eta-1})\mathbf{K}_{1}$ and $\bar{\mathbf{K}}_{2}=\eta\sigma diag(\left|\mathbf{e}\right|^{\eta-1})\mathbf{K}_{2}$. By using Lemma 2, we have

, (S.20)

where $\bar{k}_{1}=min_{j}\left\{\bar{k}_{1j}\right\},j=1,...,m$ and $\bar{k}_{2}=min_{j}\left\{\bar{k}_{2j}\right\}$ denote minimum eigenvalues of matrices $\bar{\mathbf{K}}_{1}$ and $\bar{\mathbf{K}}_{2}$, and the conditions $1<(1+\rho_{1})/2<2,\text{ }0.5<(1+\rho_{2})/2<1$ always hold. Based on the finite-time stability criterion defined in Lemma 1, the sliding surface **s** will reach zero in the following finite time:

$\frac{2^{\frac{1-\rho_{1}}{2}}V_{1}(\mathbf{x}_{0})^{\frac{1-\rho_{1}}{2}}}{\bar{k}_{1}\left(\rho_{1}-1\right)}\cdot F\left(1,\frac{\rho_{1}-1}{\rho_{1}-\rho_{2}};\frac{\rho_{1}-1}{\rho_{1}-\rho_{2}}+1;-2^{\frac{\rho_{2}-\rho_{1}}{2}}\frac{\bar{k}_{2}}{\bar{k}_{1}}V_{1}(\mathbf{x}_{0})^{\frac{\rho_{2}-\rho_{1}}{2}}\right)$. (S.21)

This ends the proof of Theorem 1.

**Appendix D. Fuzzy estimator and adaptive rules**

**Fuzzy estimator.** To approximate the functions $\mathbf{f}_{1}(\mathbf{x},t)+\mathbf{d}(t)$ and $\mathbf{F}_{2}(\mathbf{x},t)$, $m^{2}+m$ fuzzy IF-THEN rules should be designed to map the input vector $\mathbf{x}=[x_{1},x_{2},...x_{n}]^{T}\in R^{n}$ to an output$y\in R$. If the product-inference rule, singleton fuzzifier, and center-average defuzzifier are used, the output of the FLS can be defined as follows:

$y=\frac{\sum_{i=1}^{n_{r}} \tilde{y}^{i}\left(\prod_{j=1}^{n} \mu_{A_{j}^{i}}(x_{j})\right)}{\sum_{i=1}^{n_{r}} \left(\prod_{j=1}^{n} \mu_{A_{j}^{i}}(x_{j})\right)}=\mathbf{\psi}^{T}\mathbf{\zeta}(\mathbf{x})$. (S.22)

In (S.22), $n_{r}$is the total number of fuzzy rules, $\tilde{y}^{i}$ is the output at a point which $\mu_{B^{i}}(\tilde{y}^{i})=1,$ $\mu_{A_{j}^{i}}(x_{j})$ is the fuzzy membership function of the variable $x_{j}$, that is specified by Gaussian function, $\mathbf{\psi}=\left[\tilde{y}^{1},\tilde{y}^{2}\ldots,\tilde{y}^{n_{r}}\right]^{T}$ is an adjustable parameter vector, which can be regulated, and $\mathbf{\zeta}=\left[\zeta^{1},\zeta^{2},\ldots\zeta^{n_{r}}\right]^{T}$ is a fuzzy basis vector. By using the fuzzy form of (S.22), the approximation of functions $\mathbf{f}_{1}(\mathbf{x},t)+\mathbf{d}(t)$ and matrix $\mathbf{F}_{2}(\mathbf{x},t)$ are expressed as follows:

$\hat{f}_{1_{i}}(\mathbf{x},\psi_{_{f_{_{1_{i}}}}}^{t})=\psi_{_{f_{_{1_{i}}}}}^{T}\zeta_{f_{_{1_{i}}}}\mathbf{(x)}\text{, }i=1, \ldots, m$ (S.23)

(S.24)

where $\psi_{_{f_{_{i_{1}}}}}^{t}$ and $\psi_{_{f_{2_{ij}}}}^{t}$are the elements of adaptive vectors. The optimal values $\psi_{_{f_{_{i_{1}}}}}^{*}$ and $\psi_{_{f_{2_{ij}}}}^{*}$are considered as

$\psi_{_{f_{1_{i}}}}^{*}=\arg\min_{\psi_{_{f_{_{1_{i}}}}}^{t}} \left\{\sup_{x\in D_{x}} \left|f_{1_{i}}(\mathbf{x},t)+d_{i}(\mathbf{x},t)-\hat{f}_{1_{i}}(\mathbf{x},\psi_{f_{1_{i}}}^{t})\right|\right\}$ (S.25)

$\psi_{_{f_{2_{ij}}}}^{*}=\arg\min_{\psi_{_{f_{2_{ij}}}}^{t}} \left\{\sup_{x\in D_{x}} \left|f_{2_{ij}}(\mathbf{x},t)-\hat{f}_{2_{ij}}(\mathbf{x},\psi_{f_{2_{ij}}}^{t})\right|\right\}$. (S.26)

The optimal approximation error is defined as

$\mathbf{\varepsilon}_{f_{1}}(\mathbf{x},t)=\mathbf{f}_{1}(\mathbf{x},t)+\mathbf{d}(t)-\mathbf{f}_{1}^{*}(\mathbf{x},\mathbf{\psi}_{f_{1}}^{*})$ (S.27)

$\mathbf{\varepsilon}_{f_{2}}(\mathbf{x},t)=\mathbf{F}_{2}(\mathbf{x},t)-\mathbf{F}_{2}^{*}(\mathbf{x},\mathbf{\psi}_{f_{2}}^{*})$, (S.28)

and are bounded for all $\mathbf{x}\in D_{x}$ with the following inequalities:

$\left|\varepsilon_{f_{1_{i}}}(\mathbf{x},t)\right|\leq\bar{\varepsilon}_{f_{1_{i}}},\left|\varepsilon_{f_{2_{ij}}}(\mathbf{x},t)\right|\leq\bar{\varepsilon}_{f_{2}},\text{ }\forall\mathbf{x}\in\text{D}_{\text{x}}$ (S.29)

where $\bar{\varepsilon}_{f_{1_{i}}}$ and $\bar{\varepsilon}_{f_{2}}$ are positive small values.

**Adaptive rules.** To online designation of $\hat{\mathbf{f}}_{1}(\mathbf{x},\mathbf{\psi}_{f_{1}}^{t})$ and $\hat{\mathbf{F}}_{2}(\mathbf{x},\mathbf{\psi}_{f_{2}}^{t})$, adaptive rules should be extracted to adjust the parameter vectors$\mathbf{\psi}$in (S.23) and (S.24). The adaptation rules are considered as follows:

, (S.30)

, (S.31)

where $\kappa_{f_{1_{i}}}>0$ and $\kappa_{f_{2_{ij}}}>0$ are design parameters.

**Appendix E. Proof of Theorem 2**

**Part (1).** The following candidate Lyapunov function is defined as

$V=\frac{1}{2}\mathbf{s}^{T}\mathbf{s}+\frac{1}{2}\sum_{i=1}^{m} \frac{1}{\kappa_{f_{1_{i}}}}\tilde{\psi}_{f_{1_{i}}}^{T}\tilde{\psi}_{f_{1_{i}}}+\frac{1}{2}\sum_{i=1}^{m} \sum_{j=1}^{m} \frac{1}{\kappa_{f_{2_{ij}}}}\tilde{\psi}_{f_{2_{ij}}}^{T}\tilde{\psi}_{f_{2_{ij}}}+\frac{1}{2\kappa_{0}}ϒ^{2}$, (S.32)

where $\tilde{\psi}_{f_{1_{i}}}=\psi_{f_{1_{i}}}^{*}-\psi_{f_{1_{i}}}^{t}$ and $\tilde{\psi}_{f_{2_{ij}}}=\psi_{f_{2_{ij}}}^{*}-\psi_{f_{2_{ij}}}^{t}$are the estimation errors of the adapted parameters of the system. The first derivative of (S.32) is as follows:

. (S.33)

By substituting $\mathbf{u}(t)=\mathbf{u}_{eq}(t)+\mathbf{u}_{c}(t)$into (S.18), we have:

(S.34)

Equations (S.27) and (S.28) are written as follows:

$\mathbf{f}_{1}(\mathbf{x},t)+\mathbf{d}(t)-\hat{\mathbf{f}}_{1}(\mathbf{x},\mathbf{\psi}_{f_{1}}^{t})=\mathbf{f}_{1}^{*}(\mathbf{x},\mathbf{\psi}_{f_{1}}^{*})-\hat{\mathbf{f}}_{1}(\mathbf{x},\mathbf{\psi}_{f_{1}}^{t})+\mathbf{\varepsilon}_{f_{1}}(\mathbf{x})$ (S.35)

$\mathbf{F}_{2}(\mathbf{x},t)-\hat{\mathbf{F}}_{2}(\mathbf{x},\mathbf{\psi}_{f_{2}}^{t})=\mathbf{F}_{2}^{*}(\mathbf{x},\mathbf{\psi}_{f_{2}}^{*})-\hat{\mathbf{F}}_{2}(\mathbf{x},\mathbf{\psi}_{f_{2}}^{t})+\mathbf{\varepsilon}_{f_{2}}(\mathbf{x})$. (S.36)

By substituting (S.35) and (S.36) into (S.34), and multiplying $\mathbf{s}^{T}$both sides of the result, we have

(S.37)

By substituting (S.37) into (S.33), the derivative of the Lyapunov function is calculated as

, (S.38)

where

. (S.39)

(S.40)

By substituting the fuzzy adaptation rules (S.30) and (S.31) in (S.39), is obtained. Using assumption 1 and equation (22), one obtains:

$\begin{matrix} \mathbf{s}^{T}\mathbf{F}_{2}(\mathbf{x},t)\mathbf{u}_{c}(t)\geq\sigma_{0}\left\|\mathbf{s}\right\|^{2}\frac{\left|\mathbf{s}^{T}\right|\text{ }(\bar{\mathbf{\varepsilon}}_{f_{1}}+\bar{\varepsilon}_{f_{2}}\left|\mathbf{u}_{eq}\right|+\left|\mathbf{u}_{0}\right|)}{\sigma_{0}\left\|\mathbf{s}\right\|^{2}+ϒ}=\left|\mathbf{s}^{T}\right|\text{ }(\bar{\mathbf{\varepsilon}}_{f_{1}}+\bar{\varepsilon}_{f_{2}}\left|\mathbf{u}_{eq}\right|+\left|\mathbf{u}_{0}\right|)- \\ ϒ\frac{\left|\mathbf{s}^{T}\right|\text{ }(\bar{\mathbf{\varepsilon}}_{f_{1}}+\bar{\varepsilon}_{f_{2}}\left|\mathbf{u}_{eq}\right|+\left|\mathbf{u}_{0}\right|)}{\sigma_{0}\left\|\mathbf{s}\right\|^{2}+ϒ}. \end{matrix}$ (S.41)

Thus, (S.40) is bounded as follows:

. (S.42)

Using (S.41) and adaptation rule (23), it yields and

. (S.43)

Thus, all signals in the closed-loop system are bounded, and the parameters $\mathbf{\psi}_{f_{1}}^{t}$ and $\mathbf{\psi}_{f_{2}}^{t}$ converge to parameters $\mathbf{\psi}_{f_{1}}^{*}$ and $\mathbf{\psi}_{f_{2}}^{*}$ when $t\to\infty$respectively.

**Part (2).** When $\mathbf{f}_{1}^{*}(\mathbf{x},\mathbf{\psi}_{f_{1}}^{*})=\hat{\mathbf{f}}_{1}(\mathbf{x},\mathbf{\psi}_{f_{1}}^{t})$ and $\mathbf{F}_{2}^{*}(\mathbf{x},\mathbf{\psi}_{f_{2}}^{*})=\hat{\mathbf{F}}_{2}(\mathbf{x},\mathbf{\psi}_{f_{2}}^{t})$, it follows from (S.37), (S.40), and (S.43) that:

, (S.44)

where $V'=0.5\mathbf{s}^{T}\mathbf{s}$. Then, (S.44) is written as follows:

, (S.45)

where parameters $\bar{k}_{1}$ and $\bar{k}_{2}$ are the minimum eigenvalues of matrices $\bar{\mathbf{K}}_{1}$ and $\bar{\mathbf{K}}_{2}$, respectively, and $1<(1+\rho_{1})/2<2$and $0.5<(1+\rho_{2})/2<1$. Based on the finite-time stability of Lemma 1, and (S.45), the continuous nonsingular sliding surface will be reached in the finite time

$t_{s}\leq\frac{2^{\frac{1-\rho_{1}}{2}}V'(\mathbf{x}_{0})^{\frac{1-\rho_{1}}{2}}}{\bar{k}_{1}\left(\rho_{1}-1\right)}\cdot F\left(1,\frac{\rho_{1}-1}{\rho_{1}-\rho_{2}};\frac{\rho_{1}-1}{\rho_{1}-\rho_{2}}+1;-2^{\frac{\rho_{2}-\rho_{1}}{2}}\frac{\bar{k}_{2}}{\bar{k}_{1}}V'(\mathbf{x}_{0})^{\frac{\rho_{2}-\rho_{1}}{2}}\right)$. (S.46)

Therefore, the tracking errors and their first derivatives converge to zero in finite time.

**Part (3).** When $\mathbf{f}_{1}^{*}(\mathbf{x},\mathbf{\psi}_{f_{1}}^{*})\neq\hat{\mathbf{f}}_{1}(\mathbf{x},\mathbf{\psi}_{f_{1}}^{t})$ and$\mathbf{F}_{2}^{*}(\mathbf{x},\mathbf{\psi}_{f_{2}}^{*})\neq\hat{\mathbf{F}}_{2}(\mathbf{x},\mathbf{\psi}_{f_{2}}^{t})$, it follows from (S.37), (S.40) and (S.43) that:

(S.47)

or:

(S.48)

If the matrix $\mathbf{K}_{1}-diag\left(\left(\mathbf{f}_{1}^{*}(\mathbf{x},\mathbf{\psi}_{f}^{*})-\hat{\mathbf{f}}_{1}(\mathbf{x},\mathbf{\psi}_{f_{1}}^{t})\right)+\left(\mathbf{F}_{2}^{*}(\mathbf{x},\mathbf{\psi}_{f_{2}}^{*})-\hat{\mathbf{F}}_{2}(\mathbf{x},\mathbf{\psi}_{f_{2}}^{t})\right)\text{ }\mathbf{u}_{eq}\right)\times diag^{-1}(sig(\mathbf{s})^{\rho_{1}})$ is positive definite, then, (S.47) has the same structure as that of (S.44). Therefore, the finite-time stability to the boundary layer is guaranteed. By assuming

$\left(\mathbf{f}_{1}^{*}(\mathbf{x},\mathbf{\psi}_{f}^{*})-\hat{\mathbf{f}}_{1}(\mathbf{x},\mathbf{\psi}_{f_{1}}^{t})\right)+\left(\mathbf{F}_{2}^{*}(\mathbf{x},\mathbf{\psi}_{f_{2}}^{*})-\hat{\mathbf{F}}_{2}(\mathbf{x},\mathbf{\psi}_{f_{2}}^{t})\right)\text{ }\mathbf{u}_{eq}=\mathbf{W}=[W_{1},\text{ }\ldots,\text{ }W_{m}]$, if $k_{1}-\left|W_{i}\right|/\left|s_{i}\right|^{\rho_{1}}>0,$ the following region can be achieved in the finite time:

$$\left|s_{i}\right|^{\rho_{1}}\leq\frac{\left|W_{i}\right|}{k_{1}}$$

$\left\|\mathbf{s}\right\|\leq\left(\frac{\left\|\hat{\mathbf{f}}_{1}(\mathbf{x},\mathbf{\psi}_{f}^{*})-\mathbf{f}_{1}^{*}(\mathbf{x},\mathbf{\psi}_{f_{1}}^{t})\right\|+\left\|\hat{\mathbf{F}}_{2}(\mathbf{x},\mathbf{\psi}_{f_{2}}^{*})-\mathbf{F}_{2}^{*}(\mathbf{x},\mathbf{\psi}_{f_{2}}^{t})\right\|\left\|\mathbf{u}_{eq}\right\|}{k_{1}}\right)^{1/\rho_{1}}=\delta_{1}$ . (S.49)

For (S.48), similar to the analysis of (S.47), the following region is achieved in the finite time:

$$\left|s_{i}\right|^{\rho_{2}}\leq\frac{\left|W_{i}\right|}{k_{2}}$$

$\left\|\mathbf{s}\right\|\leq\left(\frac{\left\|\hat{\mathbf{f}}_{1}(\mathbf{x},\mathbf{\psi}_{f}^{*})-\mathbf{f}_{1}^{*}(\mathbf{x},\mathbf{\psi}_{f_{1}}^{t})\right\|+\left\|\hat{\mathbf{F}}_{2}(\mathbf{x},\mathbf{\psi}_{f_{2}}^{*})-\mathbf{F}_{2}^{*}(\mathbf{x},\mathbf{\psi}_{f_{2}}^{t})\right\|\left\|\mathbf{u}_{eq}\right\|}{k_{2}}\right)^{1/\rho_{2}}=\delta_{2}.$ (S.50)

By virtue of (S.49) and (S.50), the region $\left\|\mathbf{s}\right\|\leq\delta=\min(\delta_{1},\delta{}_{2})$ will be achieved in finite time. Since$\left\|\mathbf{s}\right\|\leq\delta$then $\left|s_{i}\right|\leq\delta$ $i=1,2,...,m$ and the terminal sliding surface (equation (12)) is written as follows:

, (S.51)

or, equivalently, one gets the following:

. (S.52)

When is kept, (S.52) has the same structure as the terminal sliding manifold (12). Therefore, the tracking error will converge in finite time to the following region:

$\left|e_{i}(t)\right|\leq\left(\frac{\delta}{\sigma}\right)^{\frac{1}{\eta}}$. (S.53)
